# From Thinking to Creativity: The Interplay of Mathematical Thinking Perceptions, Mathematical Communication Dispositions, and Creative Thinking Dispositions

**DOI:** 10.3390/bs15101346

**Published:** 2025-10-01

**Authors:** Murat Genç, Mustafa Akıncı, İlhan Karataş, Özgür Murat Çolakoğlu, Nurbanu Yılmaz Tığlı

**Affiliations:** 1 Department of Mathematics and Science Education, Zonguldak Bülent Ecevit University, Zonguldak 67300, Turkey; mustafa.akinci@beun.edu.tr (M.A.); ilhankaratas@beun.edu.tr (İ.K.); nurbanuyilmaz@beun.edu.tr (N.Y.T.); 2 Department of Educational Sciences, Zonguldak Bülent Ecevit University, Zonguldak 67300, Turkey; omuratcolakoglu@beun.edu.tr

**Keywords:** mathematical thinking, mathematical communication, creative thinking, structural equation modeling

## Abstract

Fostering mathematical thinking, communication, and creativity has become a central goal in mathematics education as these competencies are strongly linked to flexible problem solving and innovative engagement. Prior research has shown that students’ beliefs and dispositions play a crucial role in shaping their learning, strategy use, and persistence, yet limited evidence exists on how these constructs interrelate among pre-service elementary mathematics teachers. Addressing this gap, the present study examines the relationships among mathematical thinking perceptions, mathematical communication dispositions, and creative thinking dispositions. A correlational survey design was employed to test a hypothetical model developed within the framework of structural equation modeling (SEM). Data were collected from 615 pre-service teachers. Analyses involved descriptive statistics, correlations, and predictive algorithms via IBM SPSS Statistics 24, along with standardized regression coefficients and fit indices using AMOS. The results revealed that while perceptions of problem-solving and higher-order thinking predicted creative thinking dispositions both directly and indirectly, perceptions of reasoning did so only indirectly through mathematical communication. Mathematical communication dispositions had the strongest direct effect on creative thinking dispositions, underscoring their mediating role. These findings highlight the importance of fostering communication alongside creativity in teacher education, thereby equipping future teachers to promote creative thinking through cognitive, social, and representational processes.

## 1. Introduction

Creative thinking, one of the prominent 21st century skills, is a high-level cognitive process that enables individuals to generate original, functional, and valuable ideas, and it is gaining increasing importance in education (Beghetto, 2021; OECD, 2019; Plucker et al., 2004; Suherman & Vidákovich, 2022). In the context of mathematics education, creative thinking requires not only the emergence of individual talents but also the development of cognitive and social processes such as problem-solving, reasoning, and communication through interaction. In these processes, mathematical thinking comprising components such as problem-solving, abstraction, generalization, and logical inference provides a fundamental basis for developing creative solution strategies (Halpern, 1998; Schoenfeld, 2017; Silver, 1997; Sriraman, 2005; Stylianides & Stylianides, 2008).

It is also emphasized that creative thinking is not merely an individual mental activity, but it becomes visible and functional through the effective expression and sharing of such thoughts (Sfard, 2008). Mathematical communication encompasses individuals’ ability to clearly express their thoughts verbally, in writing, and visually, as well as the process of understanding and interpreting others’ ideas (Kaya & Aydın, 2016; National Council of Teachers of Mathematics [NCTM], 2000). Communication plays a critical role in the development of creative thinking, not only as a means of expression but also as a regulatory and transformative process (Staples & King, 2017). Research shows that in communication-based learning environments, students’ levels of creative and critical thinking significantly improve; practices such as open-ended problems, group discussions, and written explanations enhance both mathematical thinking and the capacity for creative problem-solving (Cachuela, 2023; Leikin & Elgrably, 2020; Shriki, 2010; Singer et al., 2013).

The need to better understand the interplay between mathematical thinking, mathematical communication, and creative thinking is underscored by current priorities in mathematics education. Studies emphasize that fostering mathematical thinking is not only about procedural fluency but also about cultivating flexible, reflective, and creative approaches to problem solving (Leikin & Lev, 2007; Schoenfeld, 2017). Communication, in turn, has been identified as a critical mediator of learning and creativity in mathematics, as discursive practices enable students to articulate, negotiate, and refine their ideas (Mercer & Howe, 2012; Sfard, 2008). At the same time, research shows that students’ self-perceptions and dispositions strongly shape their engagement, strategy use, and persistence in mathematical tasks (Bandura, 1997; Pajares & Miller, 1994). Despite this, limited empirical evidence exists on how pre-service elementary mathematics teachers’ perceptions of mathematical thinking are connected with their communicative tendencies and creative dispositions, particularly in the context of mathematics teacher education. Addressing this gap is crucial, as teachers’ own perceptions and dispositions are likely to influence the ways they foster these competencies in their future classrooms (Açıkgül et al., 2023).

Accordingly, the main purpose of this study is to examine the relationships among pre-service elementary mathematics teachers’ perceptions of mathematical thinking, mathematical communication dispositions, and creative thinking dispositions. Accordingly, this study contains an examination of whether perceptions of mathematical thinking significantly predict creative thinking dispositions directly and indirectly through mathematical communication dispositions using structural equation modeling. The findings of this research are expected to provide important insights into how creative thinking can be more effectively supported in educational settings by holistically explaining the interactions among these three cognitive constructs.

In line with this aim, this research seeks to answer the following questions:

- What are the levels of pre-service elementary mathematics teachers’ perceptions of mathematical thinking, mathematical communication dispositions, and creative thinking dispositions?
- What are the significant relationships among perceptions of mathematical thinking, mathematical communication dispositions, and creative thinking dispositions?
- To what extent do perceptions of mathematical thinking directly influence creative thinking dispositions, and is this relationship mediated by mathematical communication dispositions?
- How well does the proposed structural equation model fit the data in explaining the relationships among perceptions of mathematical thinking, mathematical communication dispositions, and creative thinking dispositions?

## 2. Related Literature and the Conceptual Framework for Modeling

### 2.1. Mathematical Thinking

Mathematical thinking is a multidimensional cognitive activity that comes into play as individuals comprehend mathematical concepts and apply them to various situations to solve problems (Schoenfeld, 2017; P. Singh et al., 2024). This mode of thinking encompasses a range of interrelated cognitive skills, including problem-solving, abstraction, generalization, symbolization, logical inference, and metacognitive strategies (Pourdavood & Wachira, 2015; Stylianides & Stylianides, 2008). Contemporary approaches today do not view mathematical thinking as limited to procedural knowledge; instead, they adopt a more inclusive perspective by associating it with critical thinking, creative problem-solving, and high-level cognitive awareness (Halpern, 1998; Stylianides & Stylianides, 2008). Çelik and Özdemir (2020) demonstrated that reasoning, problem-solving, and mathematical thinking are significant predictors of critical thinking. Within this framework, problem-solving emerges as a process that enables students to tackle complex problems by utilizing their knowledge and skills (Ersoy & Güner, 2014). Throughout the process, students are expected not only to perform operations but also to devise problem-specific strategies and plan and evaluate their solution paths (P. Singh et al., 2024). It may therefore be assumed that mathematical thinking assumes a subset of introspection and self-assessment as thinking qualities.

Another key aspect of mathematical thinking is reasoning, which involves individuals making logical inferences in mathematical contexts, justifying their conclusions, and establishing relationships among these conclusions to make generalizations (Lithner, 2008). Research indicates that the development of reasoning deepens students’ conceptual understanding and enables them to transfer acquired knowledge effectively to real-life situations (Mukuka et al., 2023). Moreover, students’ ability to express their thoughts and receive feedback from others contributes to their effective use of mathematical language and the restructuring of their thinking processes (Pourdavood & Wachira, 2015). In this context, higher-order thinking such as analysis, synthesis, evaluation, and metacognition has gained a central role among 21st century skills by allowing students to plan, monitor, and enhance their learning processes (Aizikovitsh-Udi & Cheng, 2015; Brookhart, 2010; P. Singh et al., 2024).

### 2.2. Mathematical Communication

Mathematical communication is defined as the process by which students express their thoughts clearly and meaningfully through verbal, written, or visual means, and comprehend and evaluate others’ explanations (National Council of Teachers of Mathematics [NCTM], 2000). This process is not limited to the externalization of individual thinking; it also encompasses the sharing and construction of knowledge through social interaction (Steinbring, 2000). Students are expected to articulate their own solution strategies, attentively observe others’ strategies, and thereby participate in mathematical thinking processes (Kaya & Aydın, 2016). Classroom interactions, discussions, feedback, and group work are fundamental practices that support this communication process (Chapin et al., 2003). Additionally, from the teachers’ perspective, mathematical communication plays a vital role in identifying students’ conceptual gaps and shaping instruction accordingly (Mooney et al., 2021; Teledahl, 2017).

Mathematical communication relies on specific sub-skills: explaining ideas, appropriately using mathematical language, understanding and evaluating others’ thoughts, and using logical and coherent language (Kaya & Aydın, 2016). The National Council of Teachers of Mathematics (National Council of Teachers of Mathematics [NCTM], 2000) aims for students to not only present their own ideas clearly through these skills but also gain the competence to analyze and, when necessary, critique others’ ideas. These skills enhance the deeper understanding of mathematical concepts while allowing students to restructure their thinking processes (Pourdavood & Wachira, 2015). Kula Ünver and Bukova Güzel (2019) showed that when pre-service teachers fail to distinguish between everyday language and mathematical language, it leads to conceptual misconceptions, whereas accurate language use supports conceptual understanding. In PISA assessment reports, this competency was included as a component of mathematical literacy between 2003 and 2012, and as of 2021, it has been defined as one of the essential 21st century skills (OECD, 2021). These developments indicate that mathematical communication is no longer merely a supportive element but has become a structural component at the core of mathematics learning (Yang, 2024).

Theoretical approaches emphasize that mathematical communication is not limited to natural language; rather, it encompasses a multimodal structure composed of symbols, representations, and sign systems (Clarke et al., 1993; Radford, 2003; Sfard, 2008). From this perspective, communication serves as a tool that links cognitive processes to external representations, functioning as an integral component of mathematical thinking and learning processes. Mathematical communication involves a multidimensional set of skills that includes the construction, expression, sharing, and interpretation of students’ ideas. The core components of this process are reading, writing, speaking, and listening (Thompson & Chappell, 2007; Yore et al., 2007). Reading refers to the process of acquiring and making sense of mathematical knowledge through symbols, graphs, formulas, and written texts (Fang & Chapman, 2020; Tang et al., 2022). Writing, on the other hand, serves as a fundamental tool that helps individuals organize their thoughts, make problem-solving processes visible, and enhance the retention of knowledge (Yusof et al., 2012). Listening encompasses the ability to comprehend others’ mathematical expressions, solution strategies, and reasoning processes (Hintz & Tyson, 2015; Hufferd-Ackles et al., 2004). Speaking enables students to articulate their thoughts verbally and refine their thinking through group discussions (Capps & Pickreign, 1993; Sfard & Kieran, 2001). These four skills are not independent of one another; rather, they exist within an interactive, integrative, and complementary structure (Yore & Tang, 2022; Yore et al., 2007). Therefore, mathematical communication is recognized as a multidimensional competence that encompasses both linguistic and visual-symbolic elements.

### 2.3. Creative Thinking

Mathematical creativity refers to the ability to generate original, meaningful, and functional ideas by utilizing mathematical knowledge, concepts, and processes (Plucker & Beghetto, 2004; Suherman & Vidákovich, 2022). The literature emphasizes that a creative product must embody both originality (novelty and uniqueness) and value (appropriateness and usefulness) (Shaw et al., 2022; Shriki, 2010). The treatment of value as a foundation for creative thinking brings along an ongoing debate in the field of mathematical creativity. Indeed, there are opinions suggesting that the value of a mathematical process or procedure may not be understood at the time of its creation, but only years later (Chamberlin & Payne, 2022). This indicates that a full consensus in the field has not yet been achieved. In this framework, creative thinking is not a skill exclusive to expert mathematicians; it also includes the cognitive process by which students, at their respective knowledge levels, develop new solution paths (Sriraman, 2009). In evaluating mathematical creativity, both the quality of the resulting product and the cognitive flexibility demonstrated during its creation are considered significant (Haylock, 1987; Sriraman, 2005). Open-ended problem-solving activities encourage students to produce solutions using diverse strategies, thereby both facilitating the observation of the creative process and enhancing non-standard thinking skills (Erez, 2004; Kattou et al., 2013).

This mode of thinking is often examined through four core cognitive skills: fluency, flexibility, originality, and elaboration (Torrance, 1974). Fluency refers to generating numerous solution ideas for a given problem; flexibility involves shifting between different approaches; originality denotes the creation of unusual and innovative ideas; and elaboration indicates the extent to which a solution is justified and explained (Kaufman & Sternberg, 2019; Sriraman, 2009). These components are essential for assessing both an individual’s creative potential and their mathematical knowledge base (Lev-Zamir & Leikin, 2013). Açıkgül et al. (2023) reported that pre-service teachers’ overall creativity levels were moderate, with fluency and flexibility dimensions also at a moderate level, while the originality dimension was low; furthermore, they found a positive and significant relationship between creativity and self-efficacy perceptions. Research also shows that creative thinking is not solely about generating new products but also involves restructuring existing knowledge and applying it in new contexts (Mann, 2006; Suherman & Vidákovich, 2022). In this regard, mathematical creativity is not only a cognitive but also an affective competency, allowing students to view mathematics not just as a field to be learned but as one to be explored and created (Craft, 2002; Sriraman, 2005).

### 2.4. Relationships Between Mathematical Thinking and Mathematical Communication

Mathematical thinking and mathematical communication are considered two complementary core skills in mathematics education (National Council of Teachers of Mathematics [NCTM], 2000). While mathematical thinking encompasses processes such as generalization, abstraction, logical reasoning, and problem-solving (Jaleel & Titus, 2016), the clear and meaningful articulation of these processes relies on mathematical communication (Kaya & Aydın, 2016). In Vygotsky’s (1978) sociocultural theory, he argues that communication is not merely an externalization of thought but also plays a constructive role in the development of thinking itself (Sfard, 2008). Accordingly, the communication process is seen as a tool that shapes individual cognitive production (Ball & Bass, 2003; Freudenthal, 1973). Research has shown that when students begin to express their thoughts, they construct and reassess these thoughts (more) clearly (Manouchehri & St. John, 2006; Pugalee, 2004). Students who are encouraged to provide written explanations tend to develop strategic and effective problem-solving approaches, and this process helps make their reasoning more visible (Gurat, 2018).

Communication not only conveys existing thoughts but also defines their quality, depth, and versatility (Yang, 2024). As students develop their abstraction and generalization, their ability to explain them through verbal, algebraic, and visual representations also strengthens (Kusumah et al., 2020). Such multiple representations enhance communication’s effectiveness and meaningfulness, while also supporting the processes of analyzing and evaluating others’ ideas. This evaluative process fosters critical thinking and reflects the depth of conceptual understanding (Costa & Kallick, 2000; Mukuka et al., 2023). Therefore, a bidirectional and cyclical relationship exists between mathematical thinking and communication: as students communicate, they clarify their thoughts, and clarified thoughts lead to more powerful forms of communication (Staples & King, 2017; Steinbring, 2000). Classroom environments that support this relationship enable deeper learning at both individual and social levels (Singer et al., 2017).

### 2.5. Relationships Between Mathematical Communication and Creative Thinking

Mathematical communication and creativity stand out in the educational vision as mutually reinforcing and inseparable components of holistic thinking (Jukić Matić & Sliško, 2024; OECD, 2019). While mathematical creativity involves the generation of original strategies, thinking through multiple approaches, and developing unexpected solutions (Guilford, 1967), communication enables these creative ideas to be shared in an organized manner through verbal, written, and symbolic means (National Council of Teachers of Mathematics [NCTM], 2000). Sfard’s (2008) definition of communication as thinking itself highlights the formative role of communication in the creative process. In this context, internal dialogue, mathematical discussions, and the exchange of ideas create an environment that enhances students’ creative thinking processes (Sawyer & Henriksen, 2024). Tasks involving idea articulation, group interaction, and the use of multiple representations promote collective creativity, thereby supporting individual creative production (Elgrably & Leikin, 2021; Liljedahl et al., 2016; Shriki, 2010).

The quality of the communication environment also plays a determining role in the emergence of creativity. In non-judgmental, supportive, and open communication settings, students feel more comfortable expressing unconventional ideas, which facilitates the development of creative thinking (Elgrably & Leikin, 2021) more than it does in judgmental environments. Verbal and written explanations, critical engagement with peers’ ideas, and multiple forms of representation (writing, visualization, symbolic expression) enrich creative cognitive interaction (Carreira & Amaral, 2018; Singer & Voica, 2017). Mathematics lessons based on discussion further support students’ abilities to generate creative problems and develop alternative solutions (Ata Baran & Kabael, 2023; Tinungki et al., 2024). Thus, communication not only conveys creative ideas but also provides a cognitive space in which these ideas are constructed and deepened (Engle & Conant, 2002). While the process of mathematical communication makes creative thinking visible, creative outputs also enhance the quality of the communication process (Leikin & Elgrably, 2020; Sfard, 2008). For this reason, their integrated development in educational programs enables students to become process-oriented and productive mathematical thinkers, and this synergy lies at the heart of modern mathematics education (Elgrably & Leikin, 2021).

### 2.6. Relationships Between Mathematical Thinking and Creative Thinking

Mathematical thinking and creativity form two foundational dimensions of mathematics education and are in strong interaction with one another (Ervynck, 2002; Haylock, 1987; Silver, 1997). While mathematical thinking encompasses cognitive processes such as problem-solving, pattern recognition, generalization, induction, and deduction, mathematical creativity emerges through the application of these processes in novel and unconventional ways (Ayvaz & Durmuş, 2021; Sadak et al., 2022; Wilkie, 2024). In this context, a study conducted by Kattou et al. (2010) demonstrated that students’ mathematical creativity can be predicted by their mathematical ability. In particular, it emphasized that inductive and deductive reasoning, as well as the ability to process similarities and differences, play a significant role in predicting mathematical creativity. Creative thinking is considered an advanced level of mathematical thinking, and generalizations formed through inductive reasoning evolve into creative propositions (Bicer et al., 2024; Ervynck, 2002), while deductive reasoning tests the validity of these propositions (Meyer, 2010). Moreover, the creative process involves not only the generation of entirely original ideas but also the meaningful recombination of existing concepts (Sriraman et al., 2011). In this process, reasoning, modeling, and problem-posing are fundamental components that support creativity (Lu & Kaiser, 2022; Singer & Voica, 2017; Wessels, 2017). Especially, recognizing patterns and forming generalizations allow students to develop creative problem-solving strategies (Jaleel & Titus, 2016). Empirical studies also support the existence of significant relationships between mathematical thinking and creativity (Bicer et al., 2021; Kattou et al., 2013).

Creative mathematical thinking processes often take shape in activities such as open-ended problem-solving, problem-posing, and generating multiple solutions, enabling students to extend routine algorithmic approaches and form new connections (Geteregechi, 2024; Singer et al., 2011). This development is linked not only to individual thinking but also to social interaction and mathematical communication (Shriki, 2010). Research on pre-service teachers shows strong links between creative problem-posing and mathematical thinking components such as abstraction and visualization (Bicer et al., 2021). In educational settings, interdisciplinary and project-based learning strategies encourage students to produce creative solutions and justify them mathematically (Voica & Singer, 2011), while articulating and defending creative ideas strengthens cognitive structuring (Singer & Voica, 2017). Therefore, making creative processes visible and assessable requires focusing not only on creative products but also on the mathematical processes driven by creative thinking (Liljedahl & Allan, 2014).

### 2.7. Potential Mediating Role of Mathematical Communication on Creative Thinking

Creative thinking is considered a core component of mathematics education, closely associated with the ability to solve complex problems, generate innovative ideas, and develop diverse perspectives (Mann, 2006; Schoenfeld, 2017; Silver, 1997; Sriraman, 2009). Mathematical thinking forms the cognitive foundation of creative thinking, encompassing high-level processes such as problem-solving, generalization, pattern recognition, and reasoning (Wilkie, 2024). However, for these thoughts to be expressed in a meaningful and systematic manner, mathematical communication plays a critical role (Kaya & Aydın, 2016; National Council of Teachers of Mathematics [NCTM], 2000; Pugalee, 2004). According to Sfard’s (2008) communicational approach to learning, the process of expressing thoughts helps construct and reshape them, laying the groundwork for the emergence of creative ideas. Research indicates that communication-based learning environments enhance creativity and critical thinking, particularly through activities such as open-ended problems, group discussions, and written explanations, which activate communication and increase the likelihood of students developing creative solutions (Leikin & Elgrably, 2020). Mathematical communication not only enables individuals to express their thoughts but also serves a regulatory function in the development of creative thinking (Elgrably & Leikin, 2021). They support processes such as evaluating diverse perspectives, restructuring ideas, and providing justification (Sfard, 2008; Staples & King, 2017). This social and cognitive interaction deepens creative thinking and serves as a bridge connecting mathematical thinking and creativity. Therefore, the effect of mathematical thinking on creative thinking can occur not only directly but also indirectly through communication. Testing this relationship using quantitative methods may offer valuable insights into the importance of integrating thinking and communication strategies in mathematics education.

### 2.8. Linking Perceptions and Dispositions

There is strong evidence that individuals’ perceptions of their own abilities, the nature of the domain, and task-related beliefs shape their behaviors and skills. From a social cognitive theory perspective, self-efficacy beliefs influence motivation, task selection, persistence, and strategy use, thereby indirectly determining performance and engagement (Bandura, 1997; Schunk & Pajares, 2002). Similarly, within self-regulated learning frameworks and motivational research, students’ awareness of and beliefs about their own cognitive processes have been shown to guide their learning strategies and problem-solving approaches (Dweck, 2006; Zimmerman, 2000). Expectancy-value approaches also argue that the perceived value of a task and perceived control shape dispositions and, consequently, behavior (Eccles & Wigfield, 2002). Moreover, within the theory of planned behavior framework, perceived behavioral control and related beliefs influence behavioral intentions, which are among the strongest predictors of behavior (Ajzen, 1991). In addition, research on creative self-efficacy demonstrates that individuals’ beliefs about their own creative capabilities predict both their engagement in and the outcomes of creative endeavors (Tierney & Farmer, 2002). Taken together, these perspectives suggest that individuals’ conceptualizations or perceptions of mathematical thinking may influence their dispositions toward creative activities via their tendencies in mathematical communication and, consequently, their intentions in practice.

In this context, existing literature often examines mathematical thinking, communication, and creativity as separate constructs, with limited attention to their integrative and mediated relationships. Especially, the role of mathematical communication dispositions as a bridge between perceptions of mathematical thinking and creative thinking dispositions has not been sufficiently explored. Furthermore, most studies have focused on secondary school students, while research involving pre-service elementary mathematics teachers remains scarce. This study addresses this gap by examining both direct and indirect effects among these three constructs through structural equation modeling, thereby offering a more comprehensive understanding of the development of mathematical creativity.

## 3. Methodology

### 3.1. Research Design

In this study, observed variable path analysis based on a correlational survey design was used to examine the predictors of pre-service elementary mathematics teachers’ creative thinking dispositions (Creswell & Creswell, 2023; Kline, 2023). The model, constructed within the framework of structural equation modeling (SEM), aims to test both the direct and indirect relationships among the variables (Byrne, 2016). Specifically, the research model consists of both exogenous and endogenous variables. The exogenous variables include four sub-dimensions of perceptions of mathematical thinking: perceptions of higher-order thinking, perceptions of reasoning, perceptions of mathematical thinking skills, and perceptions of problem-solving. These variables are assumed to influence the endogenous variables within the model. The endogenous variables comprise mathematical communication dispositions, serving as a mediator, and creative thinking dispositions, functioning as the dependent variable. The model was designed to investigate the extent to which perceptions of mathematical thinking predict creative thinking dispositions, both directly and indirectly through the mediating role of mathematical communication dispositions.

**Figure 1:**
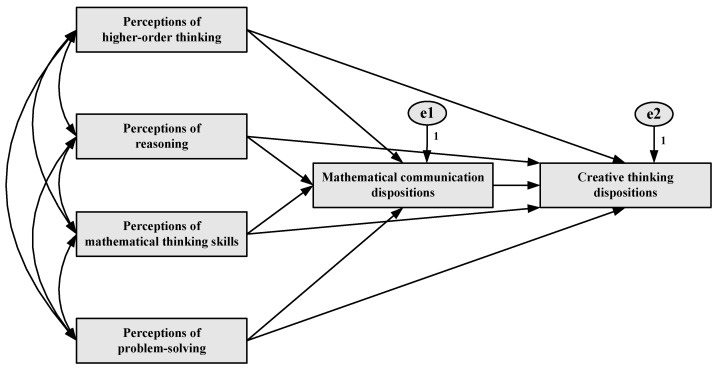
Hypothesized mediation path model for creative thinking dispositions.

The model tests the effect of students’ perceptions of mathematical thinking on their mathematical communication dispositions, as well as the indirect effects of this relationship on creative thinking dispositions. Accordingly, the initial model of this study was developed within a mediational framework grounded in the relevant literature and conceptual foundations (see Figure 1). In this model, it was hypothesized that the four sub-dimensions of perceptions of mathematical thinking would exert an indirect influence on creative thinking dispositions through mathematical communication dispositions. Moreover, the model incorporated all potential direct and indirect pathways, assuming that each sub-dimension could directly affect not only the mediating variable, mathematical communication dispositions, but also the ultimate dependent variable, creative thinking dispositions.

### 3.2. Research Context and Participants

In Turkey, the teacher training process takes place within the faculties of education through undergraduate programs in elementary and secondary mathematics education. Within the 12-year compulsory education system, graduates of the elementary mathematics education program are responsible for teaching mathematics from the fifth to the eighth grade, while graduates of the secondary mathematics education program teach mathematics from the ninth to the twelfth grade. Teacher training programs in Turkey include courses in mathematical content knowledge, pedagogical content knowledge, and instructional methods. The curriculum aims to equip teacher candidates with essential mathematical skills through subject-specific and subject-pedagogy courses. Among the subject pedagogy courses offered are “Problem Solving in Mathematics,” “Making Connections in Mathematics Teaching,” “Logical Reasoning,” “Modeling in Mathematics Teaching”, and “Communication in Mathematics Classrooms.” One of the primary objectives of the teacher education curriculum is to cultivate future mathematics teachers’ dispositions of mathematical communication, thinking, and creativity, which in turn support the development of their related skills.

The sample of this study consists of a total of 615 undergraduate students, selected based on the principle of voluntariness and using a convenience sampling method, from among the students studying in the faculties of elementary mathematics education at six different public universities in Turkey (Bartın University, Hacettepe University, Recep Tayyip Erdoğan University, Sivas Cumhuriyet University, Trabzon University, and Zonguldak Bülent Ecevit University). The dataset was analyzed using the Mahalanobis distance method to identify multivariate outliers. As a result of this analysis, 56 outliers were excluded from the dataset, and subsequent analyses were conducted based on 615 valid participants.

An examination of the participants’ demographic characteristics revealed that 74% were female (n = 455) and 26% were male (n = 160). In terms of academic year, 18.5% were first-year students (n = 114), 33% were second-year students (n = 203), 26.3% were third-year students (n = 162), and 22.1% were fourth-year students (n = 136). The distribution of students across universities was as follows: 109 at Bartın University, 83 at Hacettepe University, 31 at Recep Tayyip Erdoğan University (Rize), 132 at Sivas Cumhuriyet University, 124 at Trabzon University, and 136 at Zonguldak Bülent Ecevit University. The sample size and diversity at the academic level suggest that the sample is sufficiently robust to support the generalizability of the model tested in this study.

### 3.3. Data Collection Instruments

In this study, the data were collected using the Mathematical Thinking Scale, the Mathematical Communication Skills Scale, and the Marmara Creative Thinking Dispositions Scale. All measurement instruments used in this study are self-report, Likert-type scales. While the items in the mathematical communication skills and creative thinking dispositions scales directly capture participants’ self-reported tendencies of their competencies, the items in the mathematical thinking skills scale are phrased in terms of what constitutes mathematical thinking. As such, the latter scale reflects participants’ conceptualizations and self-endorsed perceptions of mathematical thinking rather than direct self-assessments of their own performance. In this sense, the scale captures participants’ reflective beliefs about mathematical thinking, which are theoretically linked to their self-concept and dispositions, rather than measuring actual performance-based competencies.

#### 3.3.1. Mathematical Thinking Scale (MTS)

To evaluate pre-service teachers’ perceptions of mathematical thinking, the Mathematical Thinking Scale (MTS), a Likert-type instrument developed by Ersoy and Başer (2013), was employed, comprising four dimensions: higher-order thinking dispositions, reasoning, mathematical thinking skills, and problem-solving, with a total of 25 items. In this study, it is important to note that the instrument captures participants’ perceptions of these dimensions rather than directly measuring their skills or performance. For example, a problem-solving item is “*Individuals with mathematical thinking skills try to solve problems using unconventional ways*”, while a reasoning item is “*An individual who can employ multiple reasoning approaches simultaneously is considered to have acquired mathematical thinking skills*”. Therefore, in our research model, the dimensions are presented as perceptions of higher-order thinking, perceptions of reasoning, perceptions of mathematical thinking skills, and perceptions of problem-solving, and the findings and discussion sections have been interpreted accordingly. The scale includes 20 positively and 5 negatively worded items, rated on a 5-point scale from “Strongly Agree (5)” to “Strongly Disagree (1).” Negative items were reverse-coded. The total score ranges from 25 to 125, with higher scores indicating stronger mathematical thinking. Exploratory factor analysis confirmed the four-factor structure, which explains 61.86% of the total variance. Cronbach’s alpha values for the subscales ranged from 0.73 to 0.80, indicating sufficient internal consistency (DeVellis & Thorpe, 2021; George & Mallery, 2024; K. Singh, 2007; Tavakol & Dennick, 2011; Vaske, 2019).

#### 3.3.2. Mathematical Communication Skills Scale (MCSS)

To assess pre-service teachers’ mathematical communication dispositions, a 26-item, 5-point Likert-type Mathematical Communication Skills Scale (MCSS) developed by Akıncı et al. (2025) was used. While the scale conceptualizes mathematical communication as a multidimensional skill involving reading, writing, listening, and speaking, its development process revealed a unidimensional structure. This finding indicates that mathematical communication should be evaluated as an integrated construct. The scale is one-dimensional with 26 items. In this study, it should be emphasized that the instrument does not directly assess participants’ actual communication skills; rather, it evaluates their dispositions regarding mathematical communication. For example, a writing item is “*I can represent verbal statements using tables, graphs, or written symbols when needed*”, while a listening item is “*I can understand the key points by carefully listening to a mathematical conversation*”. Therefore, in our model and throughout the presentation of findings and discussion, this construct has been treated as mathematical communication dispositions. All items are positively worded and scored from “Strongly Agree (5)” to “Strongly Disagree (1)”. The total score ranges from 26 to 130, with higher scores indicating stronger mathematical communication skills. Exploratory factor analysis explained 48% of the total variance, and confirmatory factor analysis supported the single-factor structure (χ^2^/df = 1.93; GFI = 0.82; AGFI = 0.79; CFI = 0.91; RMSEA = 0.07; RMR = 0.06), providing evidence of a well-fitting model (Browne & Cudeck, 1993; Hu & Bentler, 1999; Kline, 2023; Meyers et al., 2017; Schermelleh-Engel et al., 2003; Tabachnick & Fidell, 2019). The internal consistency reliability of the scale (Cronbach’s alpha = 0.96) indicates it is an effective tool for measuring mathematical communication skills (DeVellis & Thorpe, 2021; George & Mallery, 2024; K. Singh, 2007; Tavakol & Dennick, 2011; Vaske, 2019).

#### 3.3.3. Marmara Creative Thinking Dispositions Scale (MCTDS)

To determine individuals’ creative thinking dispositions, the Marmara Creative Thinking Dispositions Scale (MCTDS) developed by Özgenel and Çetin (2017) was used. The scale measures attitudes and orientations toward utilizing creative thinking potential. The scale consists of six subscales: innovation-seeking (8 items), courage (4 items), self-discipline (5 items), curiosity (3 items), skepticism (2 items), and flexibility (3 items), comprising a total of 25 items. For example, an innovation-seeking item is “*I use my imagination to design a new idea, artifact, or solution*”, while a flexibility item is “*I try to look at events from different perspectives*”. Items are rated on a 5-point Likert scale (1 = Never, 5 = Always), with total scores ranging from 25 to 125. Higher scores indicate a greater disposition toward creative thinking. Psychometric analyses confirmed the scale’s robustness. Exploratory factor analysis explained 55.90% of the total variance, and confirmatory factor analysis supported the six-factor structure (χ^2^/df = 2.07; GFI = 0.90; AGFI = 0.88; CFI = 0.90; RMSEA = 0.05; SRMR = 0.05), suggesting good or satisfactory model fit (Browne & Cudeck, 1993; Hu & Bentler, 1999; Kline, 2023; Meyers et al., 2017; Schermelleh-Engel et al., 2003; Tabachnick & Fidell, 2019). The internal consistency (Cronbach’s α) for the overall scale is 0.87, with subscale reliabilities ranging from 0.63 to 0.83, reflecting an adequate level of internal coherence (DeVellis & Thorpe, 2021; George & Mallery, 2024; K. Singh, 2007; Tavakol & Dennick, 2011; Vaske, 2019).

### 3.4. Validity and Reliability Analysis Results of the Scales

To assess the construct validity of the scales used in this study, Confirmatory Factor Analysis (CFA) was conducted. The analyses employed the maximum likelihood estimation method, and key fit indices such as χ^2^/df, RMSEA, CFI, GFI, and SRMR were considered to evaluate model fit. Additionally, Cronbach’s Alpha (α) coefficients were calculated to determine the internal consistency of each scale. The results for all three scales are presented in Table 1.

Reviewing the CFA results, the overall model fit of the three scales used in this study is generally acceptable. The MTS was evaluated using a four-factor model, yielding χ^2^/df = 4.57, RMSEA = 0.08, CFI = 0.75, GFI = 0.85, and SRMR = 0.09. These values, especially the CFI, suggest a limited model fit and point to potential areas for improvement. Detailed analyses revealed that the “mathematical thinking skills” factor, the third subdimension, underperformed. However, considering ethical principles and the existence of an established validity and reliability framework for the original scale, no structural changes were made, and the original four-factor version was retained. The MCSS was tested under a single-factor model, which showed a good fit with χ^2^/df = 3.50, RMSEA = 0.06, CFI = 0.90, GFI = 0.88, and SRMR = 0.05. The MCTDS was also tested using a single-factor model, demonstrating evidence of a well-fitting model (χ^2^/df = 3.20, RMSEA = 0.06, CFI = 0.88, GFI = 0.90, SRMR = 0.06). All these interpretations are based on the criteria provided by Browne and Cudeck (1993), Hu and Bentler (1999), Kline (2023), Meyers et al. (2017), Schermelleh-Engel et al. (2003), and Tabachnick and Fidell (2019).

Regarding internal consistency, Cronbach’s Alpha coefficients indicate an acceptable level of reliability for all scales. The subdimensions of the MTS (higher-order thinking dispositions, reasoning, mathematical thinking skills, problem-solving) had α values of 0.82, 0.72, 0.64, and 0.53, respectively. While the first two dimensions demonstrated strong reliability, the fourth subdimension (0.53) showed limited internal consistency; however, such a value may still be acceptable in exploratory research (DeVellis & Thorpe, 2021; Vaske, 2019). The MCSS had a high-reliability coefficient (α = 0.95), indicating excellent internal consistency. The MCTDS had an overall α of 0.90, reflecting strong reliability. Overall, the reliability coefficients confirm that the data collection instruments provided dependable measurements within the context of the research (George & Mallery, 2024; K. Singh, 2007; Tavakol & Dennick, 2011).

### 3.5. Multi-Group Confirmatory Factor Analysis

To evaluate whether the scales measured the same constructs across groups, a multi-group confirmatory factor analysis was conducted. Measurement invariance was examined sequentially at the configural, metric, scalar, structural, and strict levels. In addition to the χ^2^ difference test, criteria less sensitive to sample size, specifically ΔCFI ≤ 0.01 and ΔRMSEA ≤ 0.015, were adopted for decision-making (Chen, 2007; Cheung & Rensvold, 2002). A summary of the measurement invariance results is presented in Table 2.

For the MTS, the configural model showed acceptable fit (CFI = 0.727; RMSEA = 0.041). When factor loadings were constrained, the metric invariance model was supported (ΔCFI = 0.002; ΔRMSEA ≈ −0.001). In the full scalar model, however, ΔCFI = −0.019 exceeded the acceptable threshold. Modification Indices (MI) and Expected Parameter Change (EPC) analyses indicated that the intercept constraints of specific items (Item02, Item07, Item16, Item20) significantly impaired model fit. After releasing these intercept constraints (partial scalar model), the fit improved to ΔCFI = −0.010 and ΔRMSEA ≈ 0, supporting partial scalar invariance. At the structural level (factor covariances), ΔCFI = −0.013 indicated a slight decline in fit, while strict invariance was not supported. For the MCSS, the configural model demonstrated good fit (CFI = 0.860; RMSEA = 0.039). Metric invariance was supported (ΔCFI = 0.000), and although the χ^2^ difference test was significant for the scalar model, the practical fit indices (ΔCFI = −0.008; ΔRMSEA ≈ 0) remained within acceptable thresholds, supporting scalar invariance. At the structural level, equality of factor variances and covariances was also supported (ΔCFI = −0.001). However, the strict level exceeded the criterion (ΔCFI = −0.021). For the MCTDS, the configural model yielded acceptable fit (CFI = 0.835; RMSEA = 0.037). Measurement invariance was confirmed at the metric level (ΔCFI = 0.001, ΔRMSEA ≈ −0.002) and established at the scalar level (ΔCFI = −0.004, ΔRMSEA ≈ 0). The structural model was also confirmed (ΔCFI = 0.001), while strict invariance was not supported (ΔCFI = −0.020).

Accordingly, configural, metric, scalar, and structural invariance were established for the MCSS and MCTDS, allowing for meaningful latent mean comparisons across groups. For the MTS, full scalar invariance was not achieved; however, after MI/EPC-based adjustments, a partial scalar model was supported, permitting latent mean comparisons based on this partial scalar solution. Strict invariance was not established for any of the scales, which is a common outcome in applied measurement research (e.g., Byrne, 2016) and does not preclude the validity of latent mean analyses.

### 3.6. Data Analysis

To determine the effect of pre-service mathematics teachers’ perceptions of mathematical thinking on creative thinking dispositions through mathematical communication dispositions, the normality of the data was first examined. Mahalanobis distances were calculated to identify multivariate outliers, and participants who did not meet Mardia’s multivariate normality criterion were excluded from the analysis. The results are presented in Table 3.

The analysis showed that Mardia’s multivariate normality values were within acceptable limits. According to Byrne (2016), a Mardia coefficient below 5 indicates that the assumption of multivariate normality is met. Additionally, the skewness and kurtosis values for each variable ranged between −2 and +2, supporting the conclusion that the variables in this study satisfied the assumption of multivariate normality.

Confirmatory Factor Analysis (CFA) was also conducted for each scale used in this study to test its construct validity (see Table 1). These analyses assessed the factor structure of each scale in relation to its theoretical model, and their validity was evaluated through fit indices. Moreover, measurement invariance across grade levels was tested through multi-group confirmatory factor analyses using a stepwise approach (configural, metric, scalar, and strict). Model comparison results are summarized in Table 2.

To address this study’s first research question, mean and standard deviation values were analyzed. For the second research question, Pearson correlation coefficients (r) and significance levels were examined to assess relationships among the variables. To investigate the third research question, path analysis was conducted as part of the structural equation modeling (SEM) approach, assessing both direct and indirect effects among variables. Specifically, the effect of perceptions of mathematical thinking on mathematical communication dispositions, and the effect of mathematical communication dispositions on creative thinking dispositions were tested. The mediating role of mathematical communication dispositions in the relationship between perceptions of mathematical thinking and creative thinking dispositions was also evaluated. The AMOS software was used for model analysis, with estimations based on the maximum likelihood method. In interpreting effect sizes, the standards proposed by Cohen (2013) were employed, with β values of 0.10, 0.30, and 0.50 considered as indicative of small, medium, and large effects, respectively. In addition, to determine the statistical significance of the indirect and total effects among the variables, the bootstrap method was employed using the AMOS software. Analyses conducted with varying numbers of bootstrap resamples (200, 500, 1000, 2000, and 5000) revealed that the estimated parameters were consistent and similar across the different iterations. Accordingly, the results were reported based on the bootstrap analysis with 5000 resamples, through which bias-corrected confidence intervals were calculated to test the statistical significance of the indirect and total effects. Bootstrap is a widely preferred non-parametric technique, particularly in models involving mediation, to enhance the reliability of indirect effect estimates. An effect was considered statistically significant if the 95% confidence interval for the unstandardized or standardized estimate did not include zero. This procedure enabled the identification of significant mediation paths and provided a robust test of the total impact of each independent variable on the dependent construct. All indirect and total effect estimates, along with their corresponding *p*-values and confidence intervals, are reported in the results section. For the fourth research question, several model fit indices were used to evaluate the overall fit of the path model established within SEM. The initial evaluation of model fit was based on the chi-square statistic (χ^2^), degrees of freedom (df), and the χ^2^/df ratio. A ratio below 5 indicates an acceptable model fit (Kline, 2023; Schermelleh-Engel et al., 2003). Additional evaluation used both incremental fit indices (GFI, TLI, CFI) and error-based fit indices (RMSEA, SRMR). Values of GFI, TLI, and CFI above 0.90, and RMSEA and SRMR below 0.08 were considered indicators of acceptable model fit (Browne & Cudeck, 1993; Hu & Bentler, 1999; Meyers et al., 2017; Tabachnick & Fidell, 2019).

In the path analysis, the significance of path coefficients in the model was evaluated based on the critical ratio (C.R.) and *p*-values. A C.R. greater than ±1.96 was considered statistically significant at the 95% confidence level (Byrne, 2016).

## 4. Findings

### 4.1. Descriptive Statistics for Levels of Perceptions of Mathematical Thinking, Mathematical Communication Dispositions, and Creative Thinking Dispositions

Descriptive statistical findings related to the variables included in this study are presented in Table 4.

The mean score for perceptions of reasoning among pre-service mathematics teachers was M = 4.20, with a standard deviation (SD) of 0.53, followed by perceptions of higher-order thinking with a mean of M = 4.12 (SD = 0.50). The average scores for mathematical communication dispositions and creative thinking dispositions were relatively high at M = 4.00 (SD = 0.47) and M = 3.94 (SD = 0.46), respectively. The lowest means were observed in perceptions of mathematical thinking skills (M = 3.73, SD = 0.41) and perceptions of problem-solving (M = 3.72, SD = 0.41). Considering the scales were scored on a 1-to-5 scale, these results suggest that participants generally scored at an upper-intermediate level across all variables. The highest mean belonged to perceptions of reasoning, indicating students perceive themselves as highly competent in abstract thinking, logical inference, and reasoning processes. This is followed by perceptions of higher-order thinking, suggesting strong critical and analytical thinking dispositions. The relatively high scores for mathematical communication dispositions and creative thinking dispositions indicate positive tendencies in constructing mathematical expressions and generating innovative ideas. On the other hand, the lower means for perceptions of mathematical thinking skills and perceptions of problem-solving suggest that students may require further development in these areas. Moreover, the relatively low standard deviation values (all below 0.53) across variables indicate a homogeneous distribution of responses, suggesting that participants had generally similar levels of perceptions or dispositions.

### 4.2. Correlational Findings Among Variables

To examine the bivariate relationships among pre-service mathematics teachers’ perceptions of mathematical thinking, mathematical communication dispositions, and creative thinking dispositions, a Pearson correlation analysis was conducted. The results are presented in Table 5.

As seen in Table 5, all variables in this study showed statistically significant and positive correlations. The strongest correlation was observed between creative thinking dispositions and mathematical communication dispositions (r = 0.61, *p* < 0.01), suggesting that mathematical communication dispositions may play a crucial role in the development of creative thinking dispositions. Perceptions of higher-order thinking were significantly related to all other variables, with the strongest correlation found with perceptions of reasoning (r = 0.60, *p* < 0.01). This finding reveals that perceptions of higher-order thinking, such as abstraction and analysis, are closely linked to making logical inferences. Perceptions of mathematical thinking skills showed moderate correlations with other variables, particularly perceptions of reasoning (r = 0.46, *p* < 0.01) and perceptions of higher-order thinking (r = 0.46, *p* < 0.01). However, its correlation with mathematical communication dispositions was weaker (r = 0.26, *p* < 0.01), which may indicate that cognitive processes and mathematical communication dispositions do not always develop in parallel. Perceptions of problem-solving were significantly related to both creative thinking dispositions (r = 0.43, *p* < 0.01) and mathematical communication dispositions (r = 0.42, *p* < 0.01), indicating that perceptions of problem-solving have both creative and communicative aspects. Additionally, a positive and significant relationship was found between perceptions of mathematical thinking skills and creative thinking dispositions (r = 0.32, *p* < 0.01). This finding suggests that perceptions of mathematical thinking skills may support creative thinking dispositions. However, the comparatively lower correlation coefficient indicates that the relationship may be relatively modest compared to other variable pairs.

### 4.3. Direct, Indirect, and Total Effects

**Figure 2:**
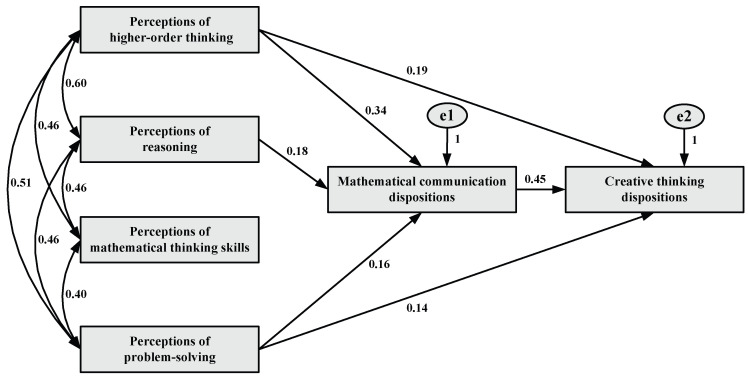
Path model of the effects of perceptions of mathematical thinking on creative thinking dispositions through mathematical communication dispositions. Note: All path coefficients in the model were found to be statistically significant from zero at the 0.001 level (two-tailed).

The final version of the model is presented in Figure 2.

The path analysis examined the direct, indirect, and total effects of the variables. The standardized values for these effects are found to be statistically significant (*p* < 0.001) and presented in Table 6.

The findings obtained through path analysis were evaluated based on the direct, indirect, and total effects of the variables influencing creative thinking dispositions. It was observed that the variables included in the model influenced creative thinking dispositions through both direct and indirect pathways. In this context, mathematical communication dispositions were found to be not only the variable with the strongest direct effect within the model but also the key mediating factor that transforms other variables into creative outputs. The standardized path coefficients and significance levels related to the model are presented in Table 6.

Accordingly, mathematical communication dispositions have a positive and high-level direct effect on creative thinking dispositions (β = 0.45, *p* < 0.001; R^2^ = 0.20). This variable does not exhibit any indirect effect, and its total effect remains at the same level (β = 0.45, *p* < 0.001). This finding indicates that approximately 20% of the variance in students’ creative thinking dispositions can be explained solely by their mathematical communication dispositions. This underscores the fundamental role of students’ tendency to express their thoughts clearly, structurally, and mathematically in the development of creative thinking.

In the model, mathematical communication dispositions function not only as a directly influential variable but also as a mediating variable that transmits the effects of other variables on creative thinking dispositions through indirect pathways. Specifically, the direct effect of perceptions of higher-order thinking on creative thinking dispositions was found to be positive yet low in magnitude (β = 0.19, *p* < 0.001; R^2^ = 0.04), accounting for approximately 4% of the variance in creative thinking dispositions. However, the indirect effect of this variable was also statistically significant (β = 0.15, *p* < 0.001, 95% CI [0.11, 0.20]), and the total effect was of moderate magnitude (β = 0.34, *p* < 0.001, 95% CI [0.26, 0.42]). This finding indicates that the contribution of perceptions of higher-order thinking to creative thinking dispositions occurs not only directly but also significantly through the mediating role of mathematical communication dispositions.

Similarly, perceptions of problem-solving exert a low-level positive direct effect on creative thinking dispositions (β = 0.14, *p* < 0.001; R^2^ = 0.02), accounting for approximately 2% of the variance in creative thinking dispositions. In addition, the indirect effect was found to be significant (β = 0.07, *p* < 0.001, 95% CI [0.04, 0.11]), and the total effect was of moderate magnitude (β = 0.21, *p* < 0.001, 95% CI [0.14, 0.29]). These results indicate that the influence of perceptions of problem-solving is substantially mediated through mathematical communication dispositions.

On the other hand, perceptions of reasoning did not exhibit a significant direct effect on creative thinking dispositions. However, its indirect effect was found to be significant (β = 0.08, *p* < 0.001, 95% CI [0.04, 0.12]), and the total effect remained at the same low level (β = 0.08, *p* < 0.001, 95% CI [0.04, 0.12]). This suggests that the influence of perceptions of reasoning on creative thinking dispositions emerges not through a direct pathway, but solely through the mediating role of mathematical communication dispositions.

The overall fit of the structural equation model was assessed using several fit indices. The model yielded a chi-square value of χ^2^(3) = 8.06 with a *p*-value of 0.05, suggesting the model fits the data significantly well. The χ^2^/df ratio was 2.69, which is below the recommended threshold of 5. Other fit indices also confirmed the model’s adequacy: RMSEA = 0.05, CFI = 0.99, GFI = 0.99, TLI = 0.98, and SRMR = 0.00. These values indicate an excellent model fit and empirical support for the theoretical structure (Browne & Cudeck, 1993; Hu & Bentler, 1999; Kline, 2023; Meyers et al., 2017; Schermelleh-Engel et al., 2003; Tabachnick & Fidell, 2019). The fact that all path coefficients were statistically significant supports the robustness and validity of the structural relationships identified. Notably, the high and significant effect of mathematical communication dispositions on creative thinking dispositions (β = 0.45, *p* < 0.001; R^2^ = 0.20) reinforces the mediating model proposed in this study.

## 5. Discussion

The findings of this study indicate that mathematical communication dispositions play a significant mediating role in the creative thinking dispositions of pre-service teachers. Structural equation modeling analyses reveal that enhanced mathematical communication dispositions significantly improve individuals’ levels of creative thinking dispositions. In particular, the sub-dimensions of perceptions of mathematical thinking, including perceptions of problem-solving and perceptions of higher-order thinking, not only exert direct effects but also influence creative thinking indirectly through mathematical communication dispositions. In other words, the tendency to effectively express, discuss, and share mathematical ideas functions as a cognitive bridge that facilitates creative thinking dispositions. Indeed, Sfard’s (2008) commognition theory underscores that thinking is fundamentally a communicative process, asserting that thinking is nothing more than internalized communication. This theoretical perspective aligns with the findings of the present study: the more effectively pre-service teachers tend to discuss and articulate mathematical concepts linguistically, the more capable they become of generating new and original ideas. In this context, a deficiency in communication dispositions may limit the potential for creative thought. Therefore, by highlighting the mediating role of mathematical communication dispositions on creative thinking dispositions, this study quantitatively supports the critical role of communication dispositions in cognitive and creative processes.

Our model demonstrates that perceptions of problem-solving and higher-order thinking positively influence mathematical communication dispositions; in turn, the development of these communication dispositions enhances creative thinking dispositions. This suggests that, for instance, a pre-service teacher who enjoys problem-solving may engage more in mathematical discussions with peers during the process, express their ideas, and advocate for different solution methods, thereby improving their communication dispositions, which, in turn, reinforces their tendency to think creatively. Thus, perceptions of problem-solving and higher-order thinking essentially form a two-stage impact mechanism leading to creative thinking dispositions. In the first stage, an individual’s tendency to engage in deep thinking and tackle challenging problems enhances their dispositions to speak, write, and discuss mathematical topics (i.e., their communication dispositions). In the second stage, these strengthened communication dispositions boost the individual’s capacity to generate new ideas and develop alternative solutions, in other words, to think creatively. This chain-effect model extends the mere practice of problem-solving in mathematics education and highlights the importance of attending to its verbal expression and communication dimension as well. A problem-solving approach that focuses solely on reaching the correct answer without incorporating communication and collaboration may fall short of making the expected contribution to creative thinking. Therefore, offering activities that not only promote problem-solving and higher-order thinking but also incorporate discussion and communicative engagement appears to be the most effective pathway for fostering the development of creativity.

As noted above, one of the direct effects identified in this study is the positive association between high levels of perceptions of problem-solving and creative thinking dispositions. This suggests that individuals who enjoy engaging with mathematical problems and who persist in seeking solutions are also highly likely to exhibit higher levels of creative thinking dispositions. Silver (1997) argued that mathematical creativity is a developable capacity and that it can be broadly cultivated in all students, particularly through rich problem-solving and problem-posing activities. Suherman and Vidákovich (2022), in their focus on the assessment and development of mathematical creative thinking in education, emphasized the indispensable role of creative thinking in problem-solving processes. Creative thinking is commonly defined as the ability to solve a problem using novel and original approaches (Kattou et al., 2013). In this study, the positive relationship between perceptions of problem-solving and creative thinking dispositions provides empirical support for this perspective. Individuals who are willing to solve problems in creative ways were found to have higher tendencies toward creative thinking. Our finding that pre-service teachers’ creative thinking dispositions increased in parallel with their perceptions of problem-solving indicates that creativity develops in close conjunction with problem-solving processes. Therefore, the results of this study empirically validate the relationships emphasized in important studies by researchers such as Sfard (2008), Silver (1997), Leikin and Elgrably (2020), and Suherman and Vidákovich (2022). Moreover, by revealing with quantitative data the mediating role of communication dispositions, this study adds a new dimension to the academic discourse.

Similarly, the understanding that pre-service teachers with strong perceptions of higher-order thinking are more willing and competent in generating creative ideas reveals that such perceptions directly contribute to creative thinking. This result is based on the idea that creative thinking does not develop through routine exercises, but rather through activities that challenge the student’s mind, prompting deep thinking and exploration of alternative approaches. Indeed, Leikin and Elgrably (2020) also emphasized that tasks given to students to foster mathematical creativity should be cognitively demanding and involve higher-order thinking processes. In this study as well, the significantly higher creative thinking scores of pre-service teachers who exhibited a tendency towards higher-order thinking support this view. A predisposition towards activities such as analyzing complex mathematical problems, comparing different solution strategies, and making generalizations also triggers a tendency to find innovative and unconventional solutions (Singer & Voica, 2017). In this way, engaging in higher-order cognitive processes effectively creates a foundation that nurtures creative thinking, and the results of this study empirically confirm this perspective. Additionally, this study’s findings show that perceptions of higher-order cognitive processes not only directly contribute to dispositions of creativity, but that creative thinking emerges even more powerfully when these processes interact with mathematical communication dispositions that allow students to express their ideas verbally, in writing, or visually.

The findings also revealed that mathematical communication dispositions are the strongest predictive variable for creative thinking dispositions. This result supports the view expressed in previous studies that there is a reciprocal and complementary relationship between communication and creative thinking (Leikin & Elgrably, 2020; Sawyer & Henriksen, 2024). Elgrably and Leikin (2021) noted that problem-posing activities based on multiple representations simultaneously enhance both communication and creativity, while Tinungki et al. (2024) found that group-based interactions increase students’ tendencies towards creative problem-solving.

Another noteworthy finding of this study is that perceptions of reasoning do not have a statistically significant direct effect on creative thinking dispositions; however, they exert an indirect effect through mathematical communication dispositions. This finding aligns with Sfard’s (2008) conceptualization of thinking as communication. Internal cognitive processes are transformed and reshaped into creative forms primarily through externalization, that is, through sharing and discussing those processes. In line with this perspective, Voica and Singer (2011) demonstrated that communicative interactions reconstruct individuals’ thinking processes and that this reconstruction plays a critical role in the emergence of creative dispositions.

Although the relatively low correlation coefficients between the constructs may at first glance suggest weak associations, they simultaneously indicate that each construct (i.e., mathematical thinking, mathematical communication, and creative thinking) retains unique, independent qualities. In other words, the constructs are conceptually distinct rather than overlapping. This distinctiveness strengthens the theoretical position of this study and contributes to the literature by highlighting the differentiated nature of these competencies.

## 6. Conclusions, Implications, and Research Directions

This study demonstrates that perceptions of mathematical thinking and mathematical communication dispositions play a decisive role in understanding pre-service teachers’ creative thinking dispositions. According to the structural equation model, perceptions of problem-solving and higher-order thinking exert both direct and indirect effects on creative thinking dispositions. On the other hand, although perceptions of reasoning are not directly effective, they significantly support creative thinking through mathematical communication dispositions. In particular, the fact that mathematical communication dispositions are the strongest predictor of creative thinking dispositions reveals that this tendency is not only a transmission tool but also a producer and organizer of creative production. This result suggests that communication should be a central component in mathematics teaching and plays an indispensable role in the development of creative thinking.

In this context, adopting systematic teaching approaches that aim to develop thinking and communication together to support creative thinking in mathematics teaching will increase students’ creative mathematical production levels (Liljedahl et al., 2016; Lu & Kaiser, 2022). Classroom environments where individual thinking processes are integrated with social interaction are the most productive contexts that support creative thinking (Elgrably & Leikin, 2021). Activities based on open-ended problem-solving, group discussions, and written explanations enable students to reveal both their intellectual flexibility and creative potential (Geteregechi, 2024; Bicer et al., 2024). In this process, teachers carefully listening to student thinking, providing feedback, and providing guidance through multiple representational tools significantly deepens the creative process (Mooney et al., 2021). Sfard (2008) argued that mathematical thinking is shaped by language and discourse, and that the act of thinking is essentially an act of communication. The findings of this study empirically support Sfard’s theoretical claim by demonstrating that individuals with strong mathematical communication tendencies exhibit higher levels of creative thinking dispositions. In light of this, two pedagogical approaches to fostering mathematical communication can be considered: one that promotes skill development organically through immersion in communication-rich environments, and another that emphasizes explicit instruction in communication strategies. While both have merit, a balanced integration of these approaches may provide the most effective support for developing students’ mathematical creativity through communication. Overall, the results highlight the critical mediating role of mathematical communication in the development of creativity and provide quantitative evidence of its impact.

The fact that the findings were obtained specifically from pre-service teachers provides important implications within the context of teacher education. Mathematical communication and creativity, both emphasized as key 21st century skills by (National Council of Teachers of Mathematics [NCTM], 2000) and OECD (2021), are fundamental dimensions that should be addressed in an integrated manner in the professional development of prospective teachers. These results underscore the importance of incorporating practices that foster creative thinking into teacher education curricula. In this regard, it is recommended that teacher education programs incorporate courses such as Mathematical Communication and Creative Problem Solving and that these courses be structured not only at the content level but also within a pedagogical context. Therefore, education policymakers and curriculum developers should revise the mathematics curriculum and teacher education content to adopt an approach that places creative thinking and mathematical communication at its core.

Since the findings of this study show that communication dispositions nurture creative thinking, they point to the need for enriching classroom communication environments in mathematics education. Providing pre-service teachers with the experience of learning through talking can lay the groundwork for their future students to learn in the same way. Likewise, developing perceptions of problem-solving and higher-order thinking in pre-service teachers can improve the quality of mathematics teaching in the long run. During their education, pre-service teachers should experience solving challenging problems that require exploration and thinking, not just routine exercises. Problem-centered learning, project-based learning, or inquiry-based activities are environments where pre-service teachers can develop both problem-solving and creativity. Teachers who are able to emphasize creativity and reflect it in their teaching practice can positively change their students’ attitudes toward mathematics and thus enable them to see mathematics as a meaningful and creative endeavor that is relevant to daily life.

The limitation of this study is that the structural model tested in this study reflects relationships derived from participants’ self-reports. While mathematical communication and creativity were assessed through items that directly capture participants’ self-assessments, the mathematical thinking scale reflects participants’ conceptualizations of what mathematical thinking entails. Future research is recommended to incorporate performance-based assessment tools (e.g., problem-solving tasks, written or oral communication activities, creative problem-solving performances) in addition to self-report scales in order to more comprehensively evaluate pre-service teachers’ skills and dispositions related to mathematical thinking, communication, and creativity, and to further explore the interaction among them through mixed-method studies supported by qualitative data conducted in diverse cultural and educational contexts and with students from various age groups. Moreover, although measurement invariance analyses confirmed that the constructs were measured equivalently across the four year levels, the possibility of learning progress across semesters cannot be ruled out. The present study focused on providing a general profile of pre-service teachers’ competencies; however, future research could adopt longitudinal or cohort-comparative designs to capture developmental differences across years of study and to explore how coursework influences these competencies over time. Furthermore, long-term, intervention-based studies aimed at tracking the co-development of creative thinking and mathematical communication may offer valuable insights for designing sustainable development strategies within instructional programs.

## Figures and Tables

**Table 1:** Confirmatory factor analysis and reliability results for the scales.

Name of Scales	Number of Factors	χ^2^/df	RMSEA	CFI	GFI	SRMR	Cronbach’s α
Mathematical thinking scale (MTS)	4	4.57	0.08	0.75	0.85	0.09	0.82; 0.72; 0.64; 0.53
Mathematical communication skills scale (MCSS)	1	3.5	0.06	0.9	0.88	0.05	0.95
Marmara creative thinking dispositions scale (MCTDS)	1	3.2	0.06	0.88	0.9	0.06	0.9

**Table 2:** Summary of Measurement Invariance Results (ΔCFI, ΔRMSEA, χ^2^ difference tests).

Scale	Metric vs. Configural	Scalar vs. Metric	Structural vs. Scalar	Strict vs. Structural	Decision
MTS	ΔCFI = 0.002 ΔRMSEA ≈ −0.001 χ^2^(51) = 43.75, *p* = 0.754	ΔCFI = −0.019 (full scalar not supported); after partial scalar, ΔCFI = −0.010 ΔRMSEA ≈ 0 χ^2^(63) = 104.32, *p* = 0.001	ΔCFI = −0.013 ΔRMSEA ≈ 0 χ^2^(42) = 94.10, *p* < 0.001	ΔCFI = −0.037 ΔRMSEA ≈ 0.001 χ^2^(96) = 244.30, *p* < 0.001	Configural + Metric + Partial Scalar
MCSS	ΔCFI = 0.000 ΔRMSEA ≈ −0.001 χ^2^(75) = 72.73, *p* = 0.553	ΔCFI = −0.008 ΔRMSEA ≈ 0 χ^2^(78) = 148.43, *p* < 0.001	ΔCFI = −0.001 ΔRMSEA ≈ 0 χ^2^(3) = 3.58, *p* = 0.310	ΔCFI = −0.021 ΔRMSEA ≈ 0.001 χ^2^(96) = 267.88, *p* < 0.001	Configural + Metric + Scalar + Structural
MCTDS	ΔCFI = 0.001 ΔRMSEA ≈ −0.002 χ^2^(72) = 67.80, *p* = 0.618	ΔCFI = −0.004 ΔRMSEA ≈ 0 χ^2^(75) = 93.98, *p* = 0.068	ΔCFI = 0.001 ΔRMSEA ≈ 0 χ^2^(3) = 0.847, *p* = 0.838	ΔCFI = −0.020 ΔRMSEA ≈ 0 χ^2^(123) = 227.86, *p* < 0.001	Configural + Metric + Scalar + Structural

Note: MTS: Mathematical thinking scale; MCSS: Mathematical communication skills scale; MCTDS: Marmara creative thinking dispositions scale. Reported Δ values are relative to the previous level of invariance. “Partial scalar” indicates that several intercept constraints were released based on Modification Indices (MI) and Expected Parameter Change (EPC) diagnostics.

**Table 3:** Normality values of the variables.

Variables	Min	Max	Skew	C.R.	Kurtosis	C.R.
Perceptions of higher-order thinking	2.17	5.00	−0.49	−4.95	0.64	3.24
Perceptions of reasoning	2.00	5.00	−0.53	−5.36	0.39	1.95
Perceptions of mathematical thinking skills	2.63	5.00	0.15	1.55	−0.04	−0.20
Perceptions of problem-solving	2.43	5.00	0.00	0.04	−0.22	−1.11
Mathematical communication dispositions	2.54	5.00	0.10	1.04	−0.02	−0.12
Creative thinking dispositions	2.36	5.00	0.00	0.04	0.19	0.94
Multivariate					1.08	1.37

**Table 4:** Descriptive statistics related to the variables.

Variables	N	Min	Max	Mean	SD
Perceptions of higher-order thinking	615	2.17	5.00	4.12	0.50
Perceptions of reasoning	615	2.00	5.00	4.20	0.53
Perceptions of mathematical thinking skills	615	2.63	5.00	3.73	0.41
Perceptions of problem-solving	615	2.43	5.00	3.72	0.41
Mathematical communication dispositions	615	2.54	5.00	4.00	0.47
Creative thinking dispositions	615	2.36	5.00	3.94	0.46

**Table 5:** The mean, standard deviation, and Pearson correlation coefficients among variables.

Variables	Mean	SD	1	2	3	4	5	6
1. Perceptions of higher-order thinking	4.12	0.5	1					
2. Perceptions of reasoning	4.2	0.53	0.60 **	1				
3. Perceptions of mathematical thinking skills	3.73	0.41	0.46 **	0.46 **	1			
4. Perceptions of problem-solving	3.72	0.41	0.51 **	0.46 **	0.40 **	1		
5. Mathematical communication dispositions	4	0.47	0.53 **	0.46 **	0.26 **	0.42 **	1	
6. Creative thinking dispositions	3.94	0.46	0.50 **	0.37 **	0.32 **	0.43 **	0.61 **	1

Note: *p* < 0.01, ** indicates statistically significant correlations at the two-tailed significance level.

**Table 6:** Structural routes, standardized values of direct, indirect, and total.

Structural Routes	Direct Effect (β)	*p*-Value	R^2^	Bootstrap 5000 CI 95%								Conclusion
				Indirect Effect (β)	CI		*p*-Value	Total Effect (β)	CI		*p*-Value	
					LB	UB			LB	UB		
PHOT → MCD → CTD	0.19	0.000	0.04	0.15	0.11	0.20	0.000	0.34	0.26	0.42	0.000	Partial Mediation
PR → MCD → CTD	-	0.000	-	0.08	0.04	0.12	0.000	0.08	0.04	0.12	0.000	Full Mediation
PPS → MCD → CTD	0.14	0.000	0.02	0.07	0.04	0.11	0.000	0.21	0.14	0.29	0.000	Partial Mediation
MCD → CTD	0.45	0.000	0.20	-	-	-	-	-	-	-	-	-
PHOT → MCD	0.34	0.000	0.12	-	-	-	-	-	-	-	-	-
PR → MCD	0.18	0.000	0.03	-	-	-	-	-	-	-	-	-
PPS → MCD	0.16	0.000	0.03	-	-	-	-	-	-	-	-	-

Note: CI: Confidence Interval; LB: Lower Bound; UB: Upper Bound; β: Coefficient (Estimate); R^2^: Squared Multiple Correlations; PHOT: Perceptions of higher-order thinking; PR: Perceptions of reasoning; PPS: Perceptions of problem-solving; MCD: Mathematical communication dispositions; CTD: Creative thinking dispositions.

## Data Availability

The data that support the findings of this study are not publicly available due to ethical restrictions related to participant confidentiality. However, the raw data supporting the conclusions of this article will be made available by the authors upon reasonable request.
